# Supplementary material for: Feedback loops in the Plk4–STIL–HsSAS6 network coordinate site selection for procentriole formation
Source: Biol Open. 2019 Sep 15;8(9):bio047175. doi: 10.1242/bio.047175 (PMC6777370; doi:10.1242/bio.047175)
Supplement: Supplementary information [file biolopen-8-047175-s1.pdf]

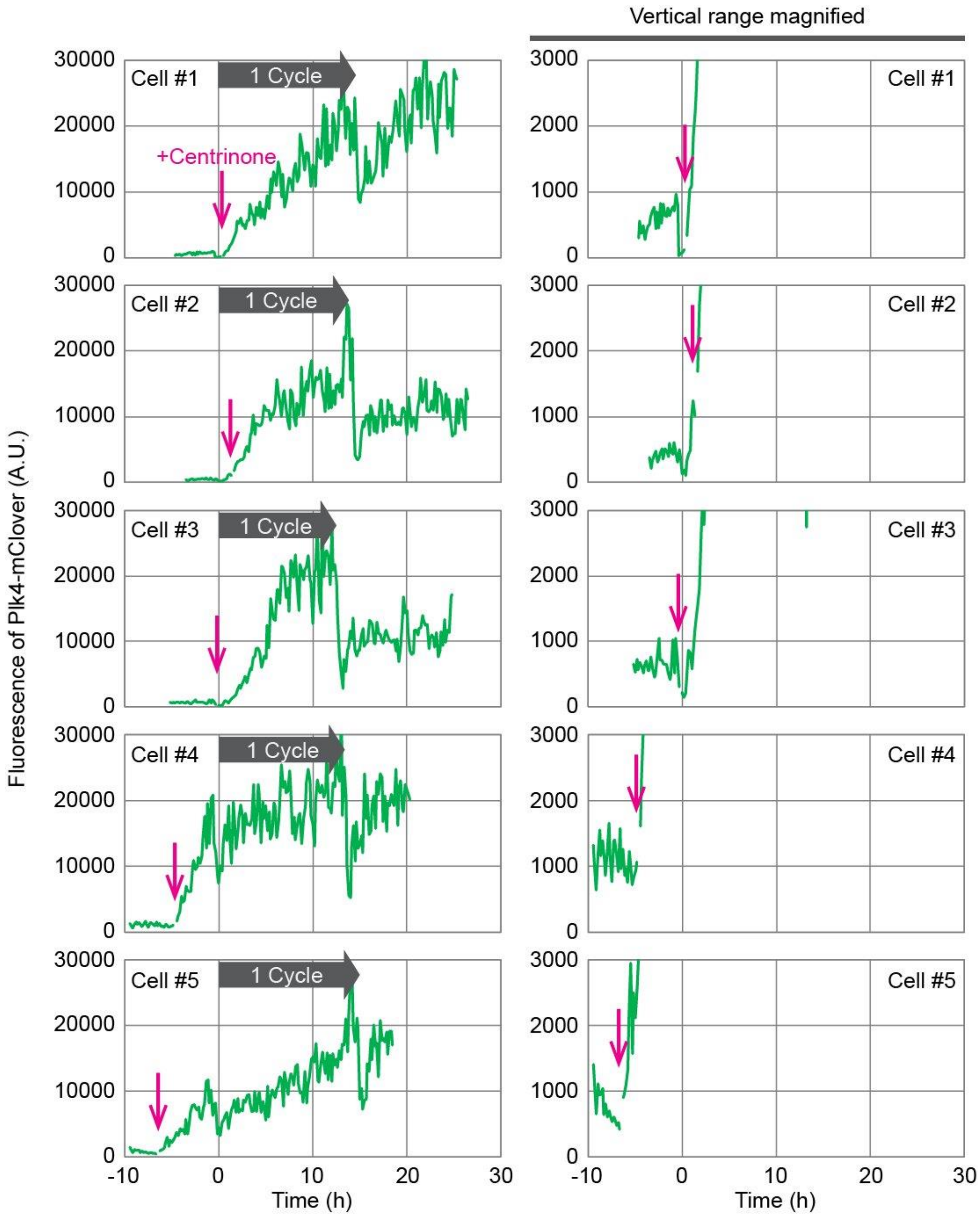

Fig S1. Effect of centrinone treatment on the centriolar accumulation of Plk4

Cells were treated with centrinone at various time points during the live imaging. Plk4-mClover signal was monitored as in Fig 1A, but centrinone was added to the culturing medium at the time points indicated by arrows. Representative graphs for five single cells are shown. As the fluorescence intensity of Plk4-mClover increased up to tenfold, the same data with an adjusted vertical range are shown in the righthand column, for comparison with the control data presented in Figs 1A and 1B. When the centrinone was added, image acquisition was paused for 20 min, as indicated by the gaps in the time course graphs.

Biology Open • Supplementary information

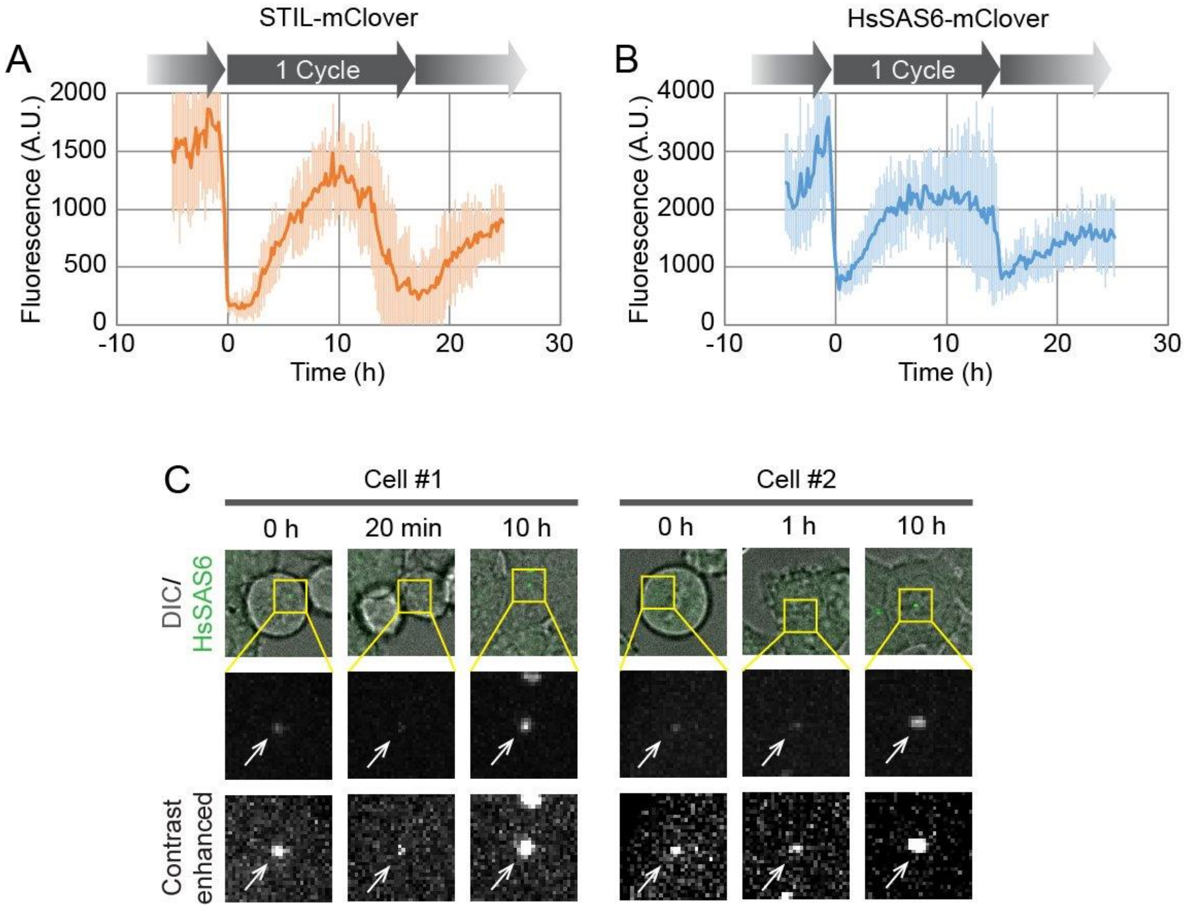

Fig S2. Live imaging of STIL and HsSAS6 endogenously tagged with mClover

(A, B) Average fluorescence of STIL-mClover (A) and HsSAS6-mClover (B). Data were obtained similarly to those in Figs 1C and 1D, except that the fluorescent tag used was mClover instead of mCherry. Graphs were prepared similarly to those in Fig 1. n = 11 cells each.

(C) Representative images for HsSAS6-mClover from two different cells. The amount of time elapsed since metaphase is shown at the top of each column. Centriolar HsSAS6-mClover is indicated with arrows in the magnified images in the central (regular contrast) and bottom (enhanced contrast) rows.

Biology Open • Supplementary information

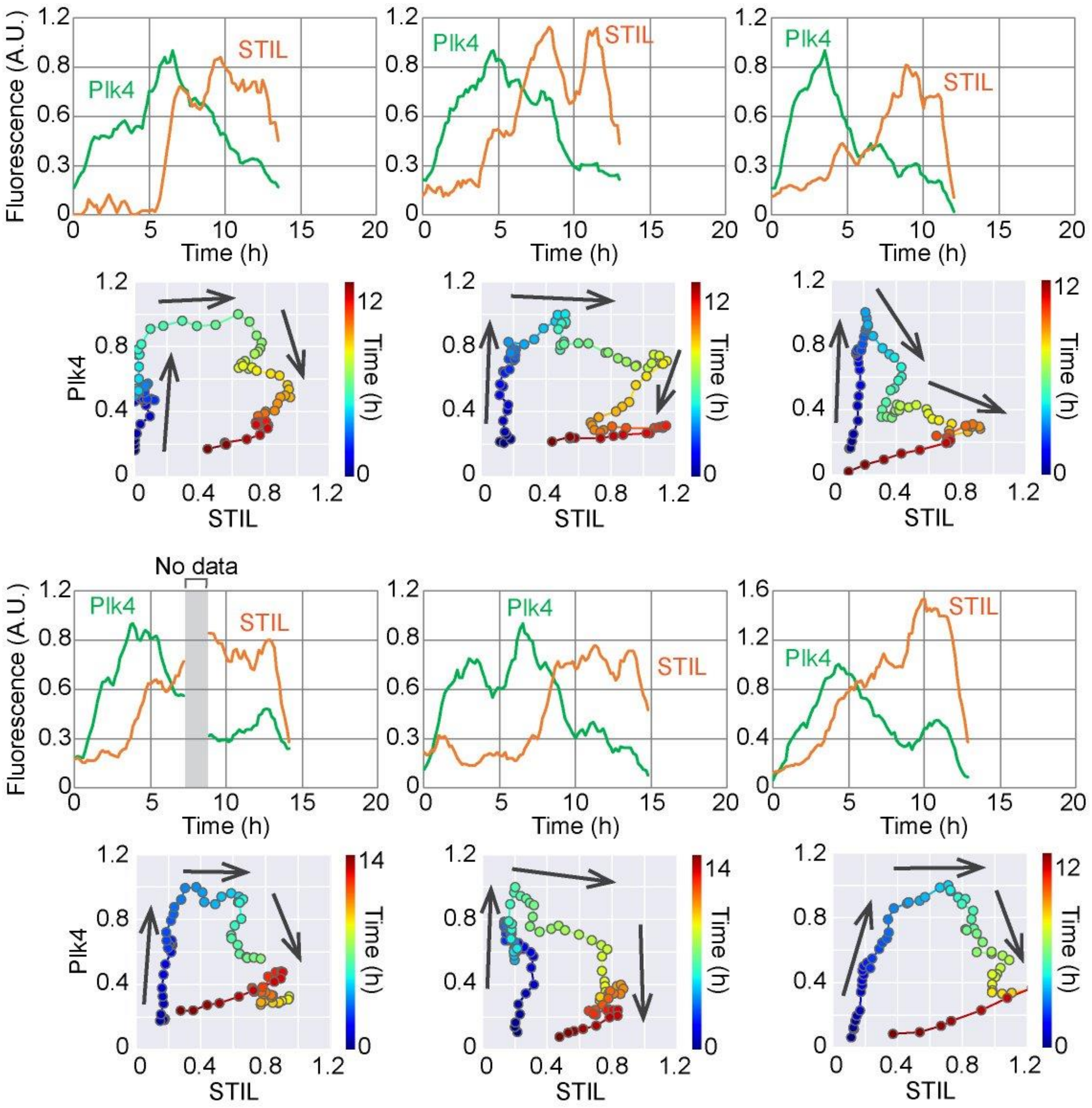

Fig S3. Simultaneous imaging of Plk4-mClover and STIL-mCherry

Example data supplementary to those presented in Fig 2A; the data for six additional cells are shown. The break in the graphs for one cell (shaded area labeled “No Data”) was due to an interruption in image acquisition caused by a bubble in the immersion oil.

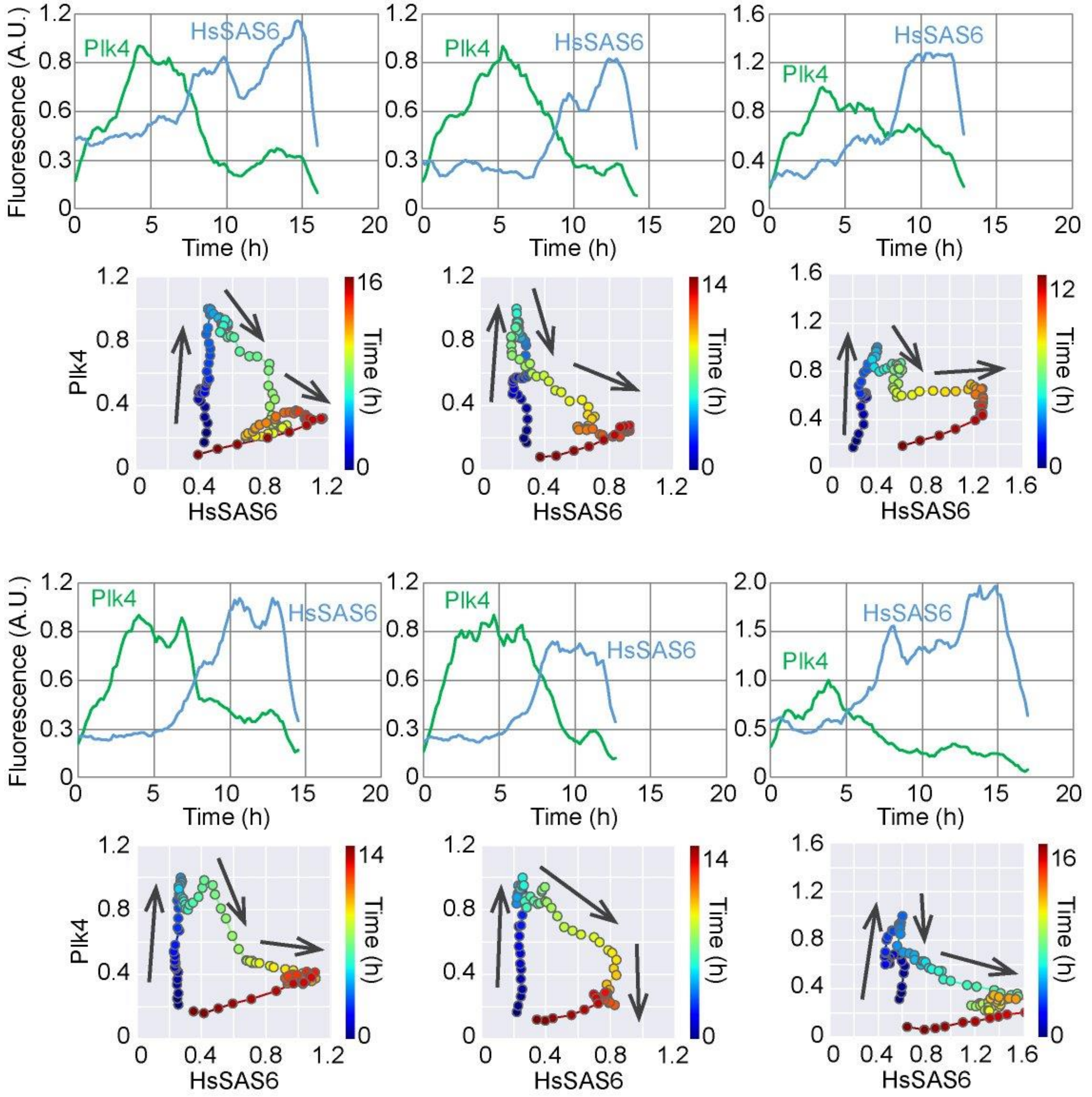

Fig S4. Simultaneous imaging of Plk4-mClover and HsSAS6-mCherry

Example data supplementary to those presented in Fig 2B; the data for six additional cells are shown.

Biology Open • Supplementary information

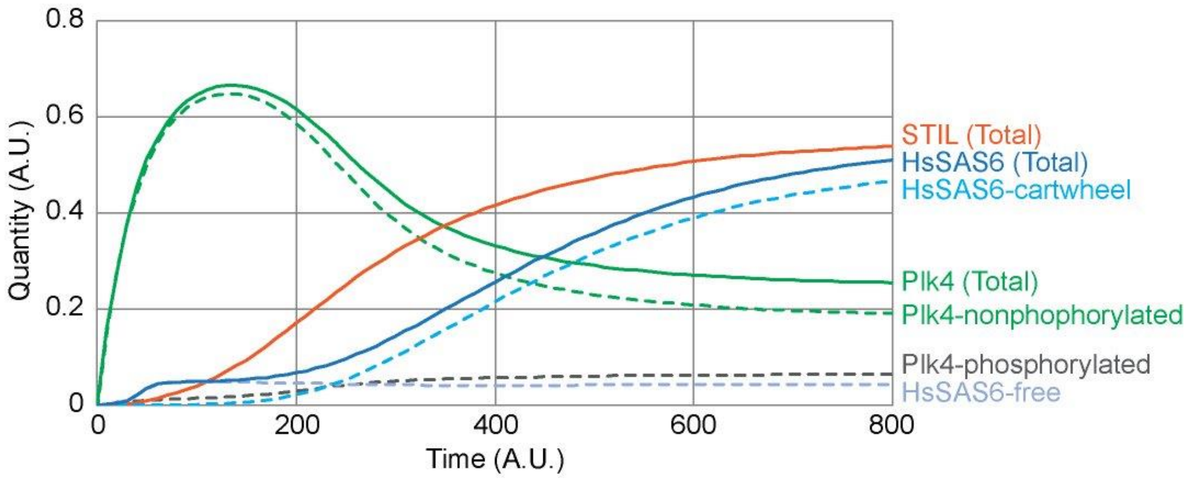

Fig S5. Simulation results for all the components modeled

A graph of the same data shown in Fig 4B showing the other components that were simulated but omitted from Fig 4B for simplicity. Specifically, the quantities of Plk4-non-phosphorylated, Plk4-phosphorylated, HsSAS6-free, and HsSAS6-cartwheel are shown here, in addition to the totals for each of the three proteins.

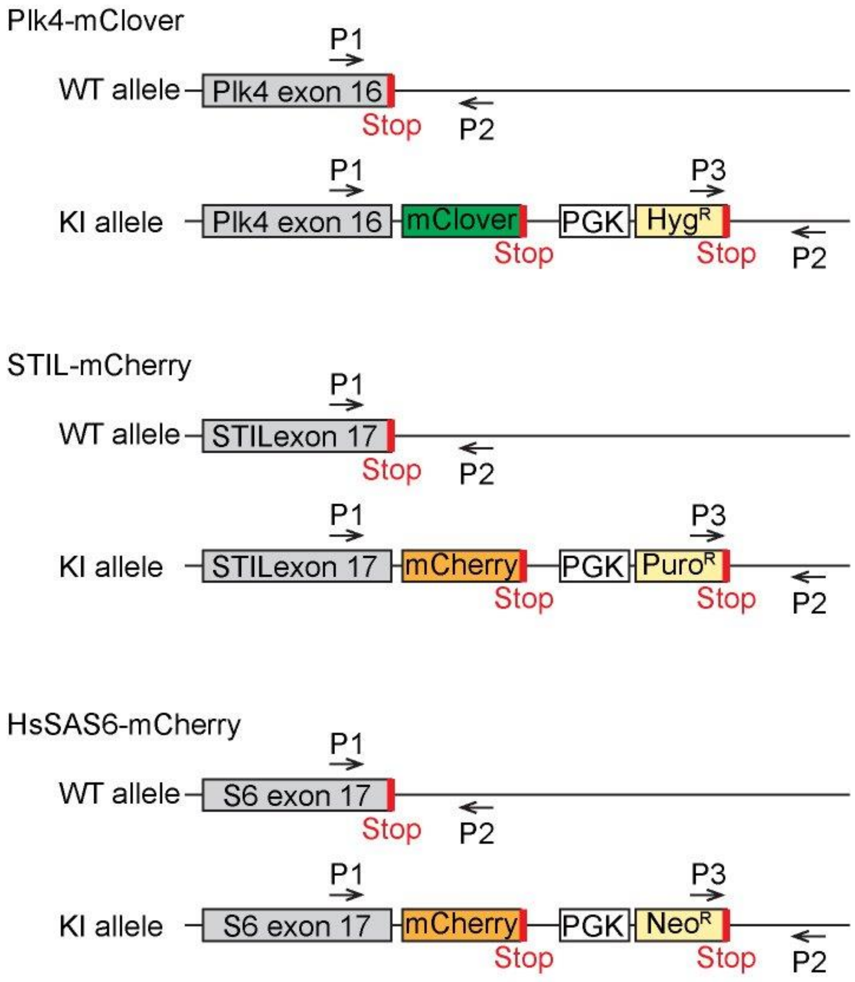

Fig S6. Endogenous tagging of the core components

The endogenous tagging system used for Plk4, STIL, and HsSAS6 are schematically shown.

Biology Open • Supplementary information
